# Case Report: B-cell–targeted therapy with ofatumumab achieves remission in refractory panuveitis and coexisting multiple sclerosis

**DOI:** 10.3389/fneur.2026.1823224

**Published:** 2026-04-29

**Authors:** Agni M. Konitsioti, Jeany Q. Lammert, Sabine Vay, Gereon R. Fink, Michael Schroeter, Clemens Warnke

**Affiliations:** 1 Department of Neurology, University of Cologne, Faculty of Medicine, University Hospital of Cologne, Cologne, Germany; 2 Department of Ophthalmology, Faculty of Medicine, University Hospital of Cologne, Cologne, Germany; 3 Cognitive Neuroscience, Institute of Neuroscience and Medicine (INM3), Research Center Jülich, Jülich, Germany; 4 Department of Neurology, University Hospital Marburg, Marburg, Germany

**Keywords:** immunotherapies, multiple sclerosis, neuro-ophthalmology, therapy refractory, uveitis

## Abstract

**Objectives/methods:**

In this case report, we present a patient with severe, refractory non-infectious panuveitis and concomitant multiple sclerosis who failed conventional immunosuppressive therapies and biologics. Non-infectious uveitis is a vision-threatening inflammatory disorder commonly driven by T-cell–mediated immune mechanisms. Increasing evidence suggests however that B cells also contribute substantially to disease pathogenesis through cytokine production, antigen presentation, and modulation of immune responses.

**Results:**

After multiple panuveitis relapses despite conventional and experimental treatment, including anti-CD52 therapy with alemtuzumab, the B-cell–depleting monoclonal antibody ofatumumab was initiated, resulting in sustained clinical remission and preservation of visual function.

**Discussion:**

This case highlights the potential role of B cells in the immunopathogenesis of non-infectious uveitis and suggests that B-cell–targeted therapy may represent an effective therapeutic option for patients with refractory disease, particularly in the context of coexisting autoimmune conditions such as multiple sclerosis.

## Introduction

Uveitis is an inflammatory disorder of the uveal tract, causing redness, pain, photophobia, floaters, and blurred vision. Although relatively uncommon, it represents a significant cause of visual impairment, accounting for approximately 10–20% of visual loss worldwide (1). Uveitis can occur at any age, but most frequently affects young and middle-aged adults (20–50 years), and shows a female predominance (2). Uveitis is defined anatomically based on the principal sites of inflammation: anterior uveitis affects the iris and ciliary body; intermediate uveitis (IU) predominantly involves the vitreous chamber; posterior uveitis affects the retina and/or choroid; and panuveitis refers to anterior, intermediate, and posterior uveitis combined (3). Etiologically, uveitis is classified as infectious or non-infectious. Infectious uveitis most commonly arises from toxoplasmosis, herpesvirus infection, tuberculosis, syphilis, or HIV, whereas non-infectious uveitis is considered to be autoimmune or immune-mediated (4).

## Pathophysiology

Experimental autoimmune uveitis (EAU) models have demonstrated that non-infectious uveitis may primarily be a T helper CD4 + Th1/Th17 cell–driven disease. Among the cytokines involved, interferon-*γ* (IFN-γ) and interleukin-17 (IL-17) are the principal mediators of Th1- and Th17-driven inflammation, respectively. Elevated concentrations of tumor necrosis factor-*α* (TNF-α) further amplify the inflammatory response by enhancing T-cell activity and macrophage activation in non-infectious uveitis. In contrast, the anti-inflammatory cytokines interleukin-10 (IL-10) and transforming growth factor-*β* (TGF-*β*), primarily secreted by regulatory T cells (Tregs), play essential roles in maintaining immune tolerance and limiting tissue damage within the eye (4).

## Uveitis and multiple sclerosis

Multiple sclerosis (MS)–associated uveitis is a rare and complex inflammatory condition that remains incompletely understood. IU is the subtype most commonly linked to MS; however, because retinal neurons are typically unmyelinated, IU is not considered a direct consequence of demyelination. Extensive cohort studies (*n* ≥ 1,000) estimate the prevalence of uveitis among patients with MS to range from 0.5 to 1.3% (mean 0.8%), whereas MS is diagnosed in 0.52 to 3.20% (mean 1.30%) of patients with uveitis (5). MS and uveitis share several genetic risk factors, including HLA-associated loci and genes encoding TNF-*α*, IL-2, and IL-6, as well as overlapping immunologic effector pathways. Elevated levels of pro-inflammatory cytokines have been detected both in CNS lesions and the vitreous, supporting the presence of a shared pro-inflammatory milieu. Consequently, it remains unclear whether uveitis represents a direct extension of MS pathology or reflects a common systemic autoimmune predisposition (6–9). This uncertainty poses significant diagnostic and management challenges for clinicians caring for patients affected by both conditions, particularly in distinguishing visual symptoms caused by demyelination, intraocular inflammation, or treatment-related complications (3).

## Treatment

Topical or systemic corticosteroids, have long constituted the cornerstone of non-infectious uveitis therapy. However, prolonged or high-dose corticosteroid use is associated with significant systemic and ocular side-effects. Current evidence-based clinical practice guidelines therefore recommend systemic corticosteroids in severe cases, in combination with conventional immunosuppressive agents such as azathioprine, methotrexate, and mycophenolate, or biologic immunomodulatory therapy (1, 10, 11). Adalimumab is a tumor necrosis factor-alpha (TNF-*α*) inhibitor, representing the only FDA−/EMA-approved biologic for non-infectious uveitis (12). For refractory disease, calcineurin inhibitors (e.g., cyclosporine or tacrolimus), infliximab, tocilizumab, or filgotinib may be considered as off-label therapeutic options (11, 13, 14). Notably, a growing body of evidence from case series supports the use of rituximab and ocrelizumab, B-cell–depleting monoclonal antibodies, for refractory non-infectious uveitis, reinforcing the hypothesis that B cells contribute to disease pathogenesis (4, 15, 16).

## Case presentation

We report the case of a male patient with a history of panuveitis and retinal vasculitis since 2002 and the diagnosis of MS since 2017, who was first referred to our department of neurology at the age of 45 years in December 2020.

At the time of the initial panuveitis diagnosis, he was 27 years old. There was no history of fever, night sweats, weight loss, prior infections, diarrhea, or symptoms suggestive of inflammatory bowel disease. The patient denied any dermatologic manifestations (such as erythema or psoriasis) or oral mucosal lesions (such as aphthae). He reported no significant joint pain, back symptoms, or family history of autoimmune disease. A chest X-ray performed in 2007 was unremarkable. Comprehensive laboratory investigations, including antinuclear antibody (ANA), rheumatoid factor (RF), anti–cyclic citrullinated peptide antibody (anti-CCP), human leukocyte antigen (HLA)-B27, soluble interleukin-2 receptor (sIL-2R), angiotensin-converting enzyme (ACE), interferon-gamma release assay (Quantiferon), as well as serology for syphilis and Borrelia, revealed no abnormalities. Thus, there was no evidence of an underlying rheumatologic, infectious, or systemic inflammatory disorder.

Initial treatment with oral prednisolone, azathioprine, and cyclosporin failed to control the uveitis activity adequately. Consequently, immunomodulatory therapy with adalimumab was initiated. The patient was treated at another ophthalmology clinic from 2014 to 2020 and received bilateral intravitreal dexamethasone implant (Ozurdex^®^) injections in March 2015 and March 2016. Due to intraocular pressure elevation, which could not be sufficiently addressed by topical and systemic anti-glaucomatous therapy, bilateral pars plana vitrectomy (ppV) with implant removal was performed in April 2016. Subsequently, in October 2016, the right eye underwent phacoemulsification with sulcus-fixated intraocular lens (IOL) implantation and repeat ppV with removal of lens fragments. The left eye remained aphakic following lens extraction, repeat ppV, and removal of residual lens material. Unfortunately, due to external treatment, details on (peri-) operative complications and the reasons for aphakia could not be clarified.

**Figure 1:**
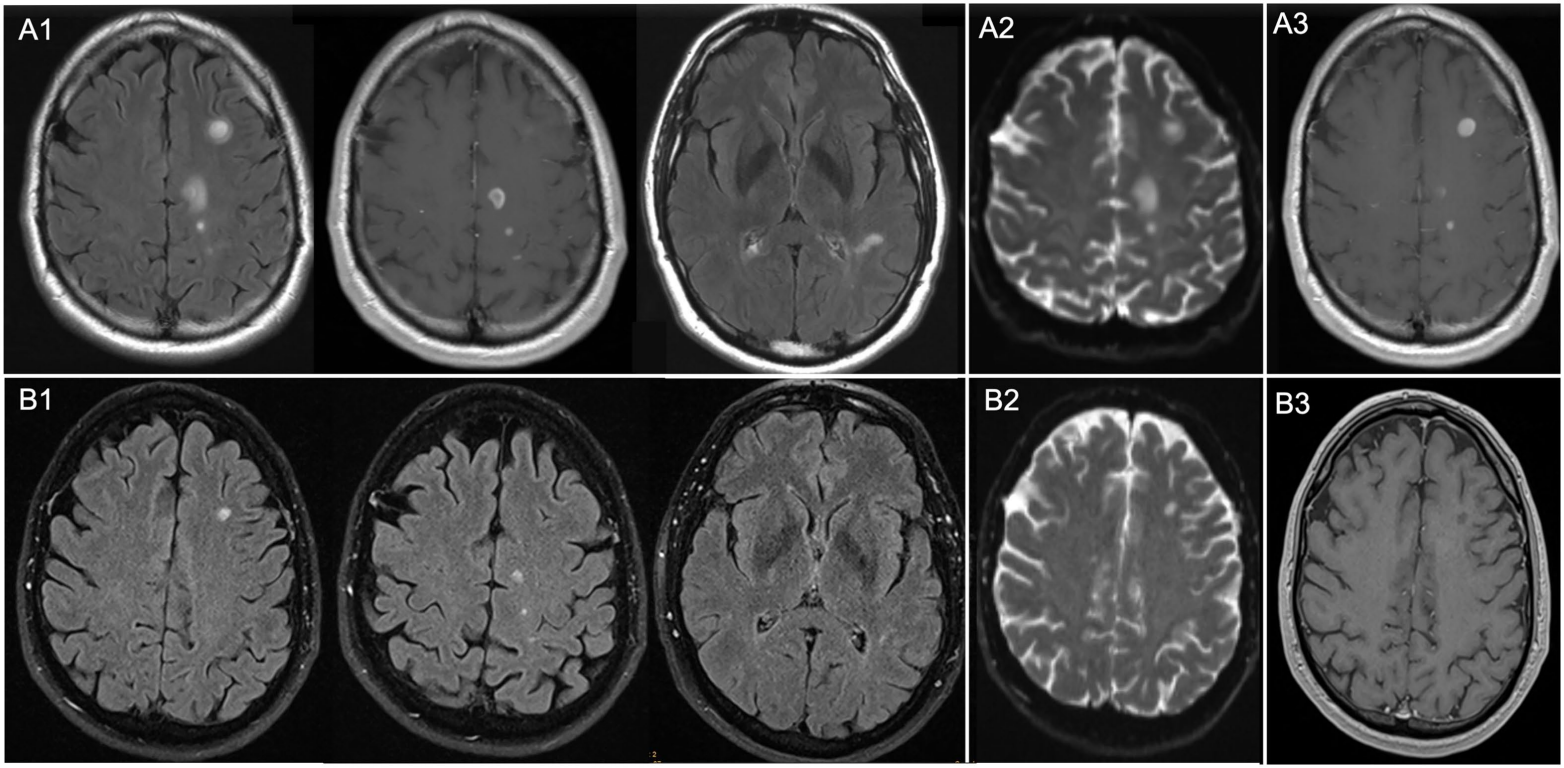
Cranial magnetic resonance imaging (MRI) scans. Top panel: cMRI from 11/2017: **(A1)** Axial fluid-attenuated inversion recovery (FLAIR) sequence demonstrating multiple hyperintense lesions, predominantly juxtacortical and temporal, consistent with an acute florid phase of chronic inflammatory CNS disease (in this case, MS). **(A2)** Diffusion-weighted MRI sequence (DWI, b = 0) demonstrating hyperintense lesions that correspond to the FLAIR-hyperintense areas. **(A3)** Gadolinium-enhanced MRI sequence illustrating contrast enhancement of the FLAIR lesions. Bottom panel: cMRI from 01/2025: **(B1)** Axial FLAIR showing no new lesions. Previously identified lesions remain broadly stable, with slight regression in some areas. No evidence of blood–brain barrier disruption or diffusion restriction is observed. **(B2)** DWI sequence (b = 0) showing only discrete, mildly hyperintense lesions. **(B3)** Gadolinium-enhanced MRI sequence demonstrating no contrast enhancement of the FLAIR lesions.

In August 2017, magnetic resonance imaging (MRI) was performed to rule out intracranial pathologies as the cause of bilateral visual impairment, revealing supra- and infratentorial cerebral lesions suggestive of MS and prompting further neurological evaluation. The case was atypical as no prior neurological symptoms suggestive of demyelination outside the visual system were reported. Nevertheless, cerebrospinal fluid analysis revealed pleocytosis and positive oligoclonal bands (type 2), while serum aquaporin-4 and myelin oligodendrocyte glycoprotein antibodies were negative, supporting the diagnosis of MS. Previous treatment with adalimumab, although reported as effective by the patient, had been discontinued after detection of cerebral lesions due to the possible risk of further exacerbating brain demyelinating disease. A follow-up MRI 3 months later (Figures 1A1–A3) showed two new lesions, suggestive of active disease, and the patient subsequently received a first cycle of alemtuzumab in December 2017. Between the first and second alemtuzumab cycles, two additional MRI lesions were reported. After the second cycle in December 2018, MRI detected no further radiological disease activity, however, the patient experienced increased uveitis activity.

**Figure 2:**
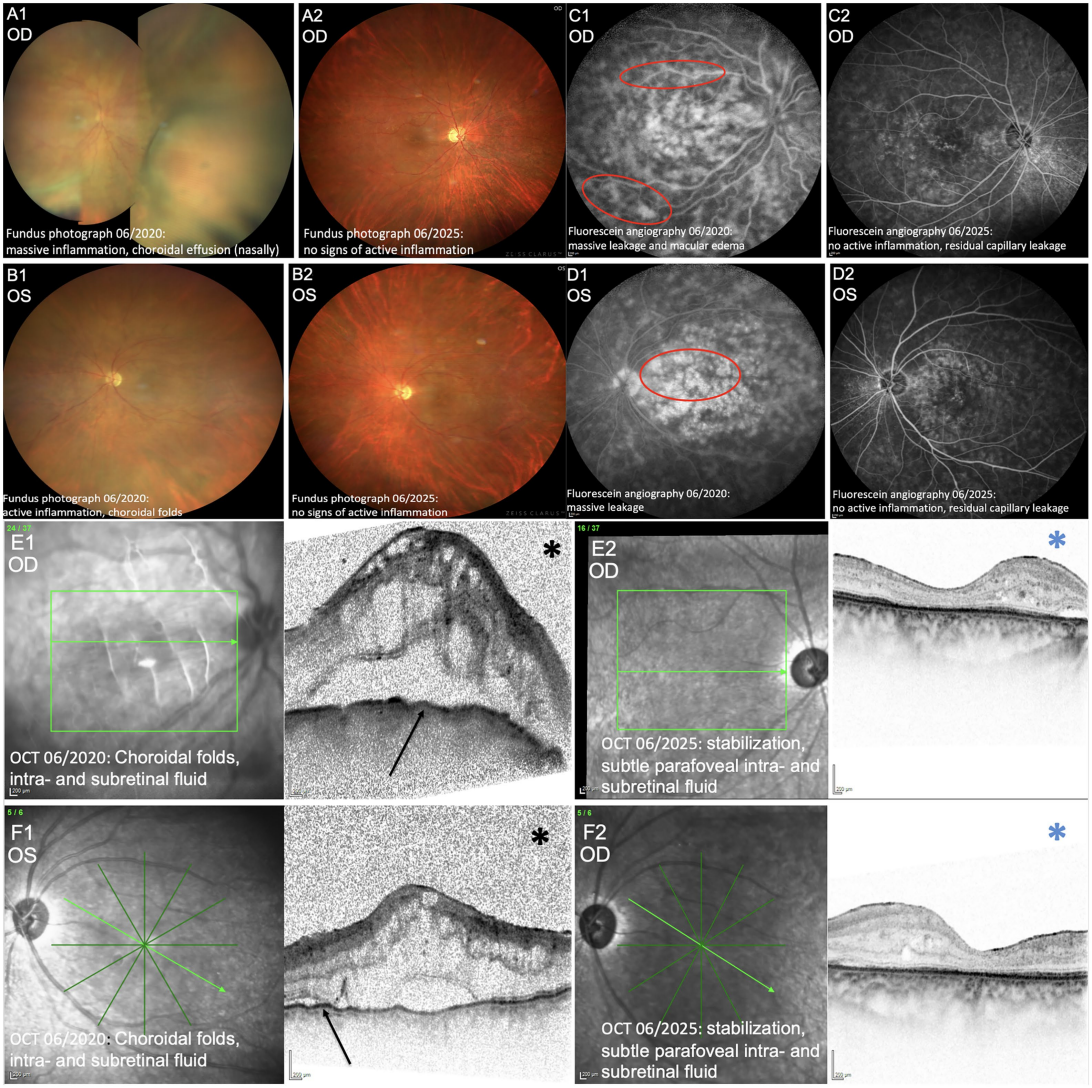
Ocular imaging findings before and after treatment. **(A1,A2,B1,B2)** Fundus photographs before treatment (06/2020, **A1,B1**) illustrate how massive inflammation and choroidal effusion cause marked haze and obscure the fundal view. Posttreatment images (06/2025, **A2,B2**) demonstrate that resolution of inflammation results in a much clearer view of the posterior pole. **(C1,C2)** Fluorescein angiography from 06/2020: macular edema, and areas of massive leakage (red circles). **(D1,D2)** Fluorescein angiography from 06/2025: No evidence of active inflammation, residual capillary leakage. **(E1,E2,F1,F2)** Optical coherence tomography (OCT) images. Pretreatment images (06/2020, **E1,F1**) demonstrate how severely active uveitis markedly reduces OCT visualization of the retina. The black asterisk indicates vitritis, which appears as a gray, snowstorm-like pattern. Black arrows highlight associated retinal thickening, choroidal folds, and intra- and subretinal fluid. Posttreatment images (06/2025, **E2,F2**) show that resolution of inflammation results in a substantially clearer OCT image, with the vitreous appearing clear (blue asterisk). OD, oculus dexter; OS, oculus sinister; OCT, optical coherence tomography.

The patient presented to our department of ophthalmology in 2020. Ophthalmological examination in 06/2020 revealed markedly reduced visual acuity (oculus dexter [OD] 1.7 logMAR; oculus sinister [OS] 0.8 logMAR), ocular hypotony (OD 2 mmHg, OS 4 mmHg), pale optic discs (Figures 2A1,A2,B1,B2), macular edema (Figures 2E1,E2,F1,F2), and bilateral venous, non-occlusive retinal vasculitis on fluorescein angiography (Figures 2C1,C2,D1,D2). Systemic therapy with high-dose oral corticosteroids (prednisolone 100 mg/day) was initiated, in combination with topical therapy, including ketorolac trometamol, and topical corticosteroids.

By August 2020, visual acuity had improved (OD 1.0 logMAR, OS 0.6 logMAR) with regression of macular edema, while systemic oral corticosteroids were tapered. However, in December 2020, the patient had experienced deterioration, particularly in the right eye, with anterior and intermediate uveitis, glaucomatous cupping, and worsening macular edema. After presentation to our neurology department in December 2020, adjunctive therapy with interferon-*β*1b was recommended to address both MS and uveitis.

From January to March 2021, corticosteroids were temporarily increased to control inflammation during beta-interferon initiation, with gradual tapering and adjustment of topical therapy. By September 2021, ophthalmologic status stabilized under low-dose oral corticosteroids (7.5 mg/day) and beta-interferon, with continued ocular topical treatment. In June 2022, new-onset anterior chamber inflammation prompted intensification of systemic corticosteroid and local therapy.

In August 2022, a second course of azathioprine was initiated for persistent uveitis, and beta-interferon was discontinued. Follow-up through May 2024 showed fluctuating inflammation, visual acuity ranging from OD 1.0–0.9 logMAR and OS 1.3–0.6, and OCT findings of persistent but regressing macular edema. Systemic therapy included maintenance corticosteroids (7.5 mg), and topical treatment was maintained and adjusted as needed. Throughout this period, cranial MRI remained stable with no new lesions, and the patient experienced no clinical MS relapse.

**Figure 3:**
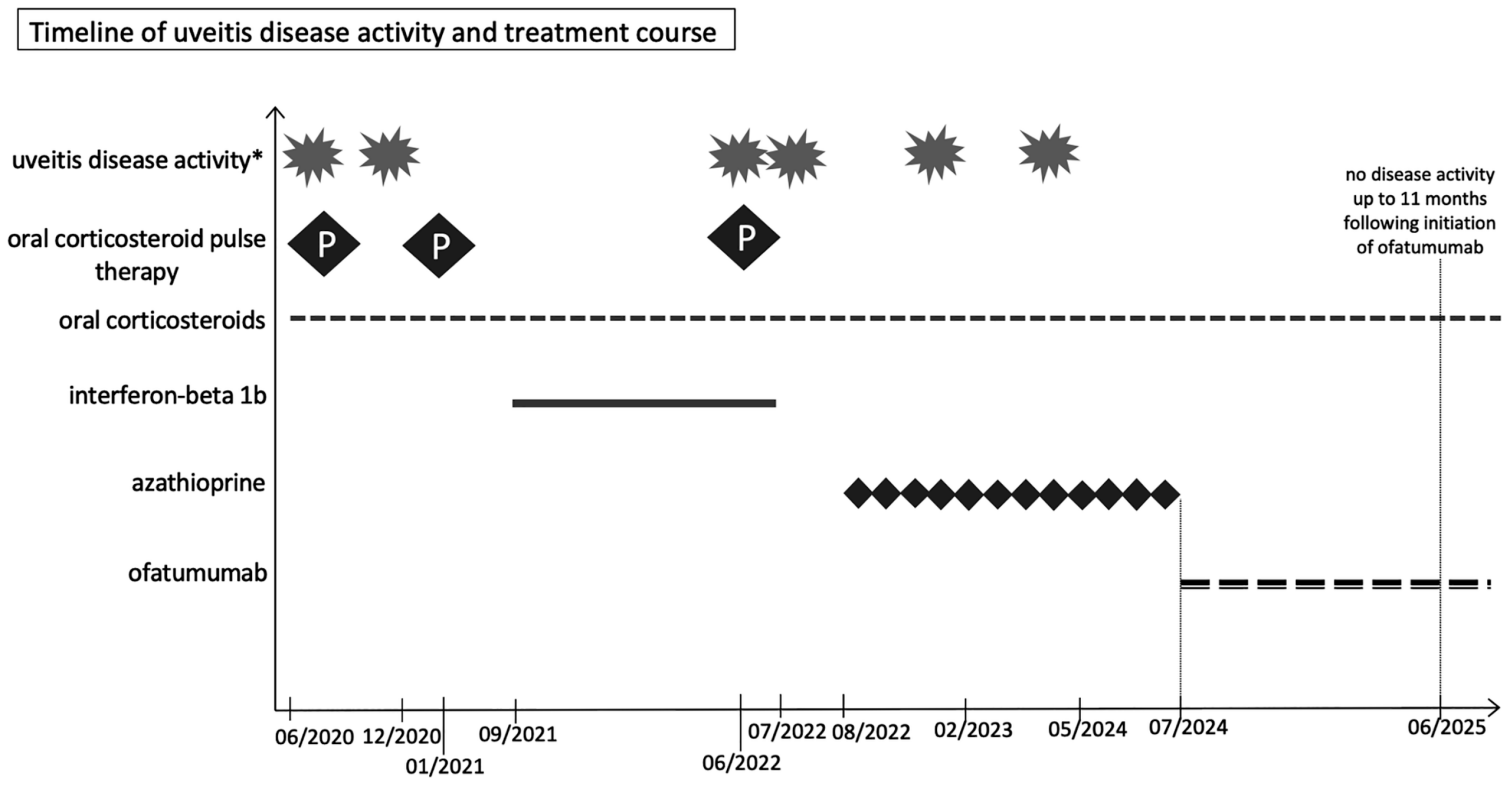
Illustration of the clinical progression, including treatment interventions and uveitis disease activity over time. *: Including clinical and paraclinical signs of disease activity as documented by fundoscopy, optical coherence tomography, and fluorescein angiography.

Given fluctuating inflammation and the risk of vision loss, azathioprine was discontinued and, after interdisciplinary discussion, immunotherapy was escalated to a B-cell–depleting treatment with ofatumumab, which was initiated in July 2024. Follow-up in November 2024, March 2025, and June 2025 demonstrated stable visual acuity (OD 1.0 logMAR, OS 0.7 logMAR) and intraocular pressure as well as controlled inflammation on OCT (Figures 2E2,F2) and fluorescein angiography (Figures 2C2,D2), under low-dose systemic corticosteroids (7.5 mg) and monthly ofatumumab. Routine follow-up cMRI continued to show no new lesions (Figure 1B), confirming stable MS without relapses, and the patient remained under close ophthalmologic and neurological monitoring. A graphical representation of the clinical progression, treatment interventions, visual acuity, intraocular pressure, and disease activity over time is provided in Figure 3 and Supplementary Table 1.

## Discussion

This case report demonstrates sustained disease control in a patient with severe, treatment-refractory non-infectious panuveitis and coexisting MS following initiation of ofatumumab after failure of multiple prior immunotherapies. Although the patient did not present with a typical demyelinating isolated syndrome as required by the 2017 McDonald criteria (17), the diagnosis of MS was supported by MRI demonstration of dissemination in space and time, cerebrospinal fluid–specific oligoclonal bands and exclusion of alternative diagnoses. The 2024 McDonald criteria (18), which accommodate atypical presentations, may be more applicable to this case, according to which the MS diagnosis can be established. Few studies have specifically examined the impact of established treatments in patients with coexistent uveitis and MS. Because many immunotherapies for MS and uveitis target the same effector cell populations and/or influence leukocyte trafficking from the blood to the CNS, these agents hold considerable potential in managing patients with both conditions.

Interferon alfa (IFN-*α*) is effective in treating uveitis, particularly in Behçet disease. However, evidence supporting the efficacy of IFN-*β* in uveitis is weaker (10). Isolated case reports and retrospective studies have described improvement in patients with uveitis who were coincidentally started on glatiramer acetate or mycophenolate mofetil (MMF) for MS; however, the evidence base for MMF is substantially stronger in uveitis than in MS (19, 20). Azathioprine has also been used in both diseases, although supporting data remain limited. Importantly, platform therapies are no longer considered adequately effective for inflammatory active forms of MS, with substantial and growing evidence demonstrating the superior efficacy of newer compounds, including natalizumab, sphingosine-1-phosphate receptor modulators such as fingolimod, or B-cell–depleting agents (21). EAU models support the use of fingolimod during active uveitis. Still, human clinical trial data are lacking, and potential benefits must be weighed against known ocular risks such as macular edema (reported in approximately 0.2% of treated individuals) (22). Additionally, relapse rates are higher in patients with MS and concomitant uveitis treated with fingolimod (22). In one patient with tumefactive MS, natalizumab treatment led to near-complete resolution of coincident intermediate uveitis (23). Alemtuzumab, which reduces activation of peripheral effector T cells, has been reported to improve treatment-refractory uveitis but is now rarely used in MS due to safety restrictions (secondary autoimmunity, vascular events) and changes in prescribing guidance (24). Remarkably, our patient demonstrated refractory uveitis despite alemtuzumab therapy, indicating that lasting remission following this therapy cannot be achieved in all patients.

Although anti-TNF agents showed efficacy in EAE, clinical trials paradoxically demonstrated worsening and induction of demyelinating events in patients with MS (25), explaining why these agents are not used in uveitis and concomitant MS Similarly, cases of CNS demyelination have been reported in patients with rheumatoid arthritis treated with tocilizumab (26).

Of particular note, differential diagnoses of uveitis with concomitant white matter lesions, including sarcoidosis, Behçet disease, other demyelinating disorders (such as neuromyelitis optica spectrum disorder and myelin oligodendrocyte glycoprotein antibody–associated disease), infectious etiologies, and neoplastic syndromes such as primary intraocular lymphoma, should be carefully considered and excluded in atypical or treatment-resistant cases.

### B-cell role in non-infectious uveitis

Non-infectious uveitis has traditionally been regarded, largely on the basis of animal models and supported by limited human evidence, as a T-cell–mediated disease, while the precise contribution of B cells to its pathogenesis remains incompletely understood (27). However, accumulating evidence from both experimental models and human studies indicates that B cells play a significant role in disease modulation, encompassing both pro-inflammatory and anti-inflammatory functions. In most EAU models, only a small proportion of B cells and plasma cells infiltrate inflammatory lesions—for example, one study reported a ratio of approximately 1:4 between CD19^+^ B cells and CD4^+^ T cells. However, increases in B-cell numbers correlate with prolonged disease duration and greater inflammatory severity (28). Another critical pathological feature linked to B-cell activity is the formation of ectopic lymphoid structures (ELSs). These structures can arise in chronically inflamed tissues, including the joints in rheumatoid arthritis, the central nervous system in MS, and the uvea in chronic non-infectious uveitis (29, 30). ELSs, primarily composed of B cells, may contribute to increased disease severity and local autoantibody production in non-infectious uveitis (4). Conversely, regulatory B cells (Bregs) function primarily by secreting anti-inflammatory cytokines, including IL-10, IL-35, and TGF-*β*. EAU models demonstrate that both the frequency and function of Bregs are significantly reduced during active inflammation, a deficiency that correlates with heightened Th1 and Th17 responses, increased production of pro-inflammatory cytokines, and more severe tissue damage (31). A noteworthy observation is that, in our patient, alemtuzumab therapy, which depletes both T and B cells, resulted in effective control of MS but failed to control the uveitis. The latter finding may be attributable to the relatively rapid B-cell repopulation after alemtuzumab in contrast to the sustained depletion achieved with anti-CD20 therapies (32).

### B cell depletion therapy

In the context of non-infectious uveitis, rituximab, an anti-CD20 monoclonal antibody, has demonstrated efficacy in a variety of refractory conditions, including chronic anterior uveitis, Behçet disease–associated uveitis, juvenile idiopathic arthritis–associated uveitis (33) and Vogt–Koyanagi–Harada disease (34). B-cell–depleting therapy with rituximab appears to alleviate ocular inflammation through autoantibody-independent mechanisms (4) by eliminating pro-inflammatory B cells, thereby reducing T-cell activation and the associated cytokine release. Simultaneously, it may restore both the quantity and function of the Breg compartment, which is critical for maintaining peripheral immune tolerance. Moreover, rituximab can disrupt the formation of ELSs, which are often associated with chronic inflammation and local autoantibody production. In this case, it remains difficult to determine whether uveitis represented a direct manifestation of multiple sclerosis and whether its remission was secondary to overall disease stabilization. Notably, apart from uveitis, there was no evidence of MS activity following discontinuation of adalimumab and completion of two cycles of alemtuzumab, suggesting that MS remission may have been achieved prior to initiation of ofatumumab.

## Conclusion

This case highlights the interplay between autoimmune demyelinating disease and non-infectious uveitis and underscores the emerging role of B cells in chronic, treatment-refractory ocular inflammation. The patient had recurrent panuveitis and MS with only limited responses to conventional immunosuppression and T-cell– or cytokine-targeted biologics. In contrast, B-cell depletion with ofatumumab led to sustained disease stabilization, supporting B-cell modulation as a promising strategy for refractory non-infectious uveitis, particularly in patients with coexisting autoimmune disease. As established, on-label, and well-tolerated therapies for MS, ofatumumab, ocrelizumab and ublituximab may represent more suitable options than off-label agents such as rituximab in patients with concurrent MS and uveitis. Close multidisciplinary collaboration between ophthalmologists and neurologists remains essential to optimize treatment outcomes in this patient population.

## Data Availability

The datasets presented in this article are not readily available because of ethical and privacy restrictions. Requests to access the datasets should be directed to the corresponding author.

## Glossary

IU: intermediate uveitis
EAU: Experimental autoimmune uveitis
MS: multiple sclerosis
IFN-*γ*: interferon-γ
IL-17: interleukin-17
IL-6: interleukin-6
TNF-a: tumor necrosis factor-*α*
TGF-*β*: transforming growth factor-*β*
Tregs: regulatory T cells
CNS: central nervous system
MTX: methotrexate
MMF: mycophenolate mofetil
ANA: antinuclear antibody
RF: rheumatoid factor
anti-CCP: anti–cyclic citrullinated peptide antibody
HLA: human leukocyte antigen
sIL-2R: soluble interleukin-2 receptor
ACE: angiotensin-converting enzyme
ppV: pars plana vitrectomy
IOL: intraocular lens
RRMS: relapsing–remitting multiple sclerosis
MRI: Magnetic Resonance Imaging
OD: oculus dexter
OS: oculus sinister
SD-OCT: Spectral-domain optical coherence tomography
IFN-a: Interferon alfa
EAE: experimental autoimmune encephalomyelitis
ELS: ectopic lymphoid structures
Bregs: regulatory B cells
JIA: juvenile idiopathic arthritis
VKH: Vogt–Koyanagi–Harada
