# Supplementary material for: Case Report: B-cell–targeted therapy with ofatumumab achieves remission in refractory panuveitis and coexisting multiple sclerosis
Source: Front Neurol. 2026 Apr 29;17:1823224. doi: 10.3389/fneur.2026.1823224 (PMC13167418; doi:10.3389/fneur.2026.1823224)
Supplement: Supplementary file 1 [file Table_1.DOCX]

**Supplementary Table 1.**

| **Date** | **Visual Acuity (OD / OS) decimal and logMAR** | **Intraocular Pressure (OD / OS)** | **Ophthalmologic Findings** | **Systemic Therapy** | **Topical Therapy** |
| --- | --- | --- | --- | --- | --- |
| **June 2020** | 0.02 / 0.16 (1.7 / 0.8 logMAR) | 2 / 4 mmHg | Bilateral old speckled endothelial deposits; pale optic discs; macular edema; attached retinas; severe bilateral retinal vasculitis (FA); intraretinal fluid (SD-OCT) | Oral corticosteroids (Decortin H 100 mg/day → taper); Pantoprazole 20 mg/day | Dorzolamide 2×/day; Ketorolac 2×/day bilaterally; Brimonidine 2×/day OD |
| **August 2020** | 0.1 / 0.25 (1.0 / 0.6 logMAR) | not specified | Quiescent anterior segment; minimal vitreous flare; healthy optic discs; regression of macular edema (SD-OCT) | Decortin H 40 mg/day → tapered weekly to 7.5 mg/day; Pantoprazole 20 mg/day | Dorzolamide 2×/day; Ketorolac 2×/day bilaterally; Brimonidine 2×/day OD |
| **December 2020** | 0.05 / 0.2 (1.3 / 0.7 logMAR) | 9 / 12 mmHg | Active anterior & intermediate uveitis, 2–3+ anterior chamber cells, 1–2+ vitreous cells, glaucomatous cupping; macular edema; intraretinal/subretinal fluid increased | Decortin H 15 mg/day → taper to 7.5 mg/day | Dorzolamide 2×/day; Ketorolac 2×/day; Lotemax® 5×/day; Brimonidine 2×/day OD |
| **January 2021** | 0.1 / 0.25 (1.0 / 0.6 logMAR) | 13 / 8 mmHg | Mild residual anterior chamber inflammation OS; quiescent OD | Decortin H 7.5 mg/day + temporary pulse increase (100 → 50 → 25 → 10 mg/day); Betaferon® SC 3×/week | Lotemax® hourly → tapered; Dorzolamide, Ketorolac, Brimonidine OD maintained |
| **March 2021** | 0.1 / 0.25 (1.0 / 0.6 logMAR) | 15 / 12 mmHg | Quiescent anterior segment; regression of macular edema OS | Decortin H 10 mg/day; Pantoprazole 20 mg/day | OD: Brimonidine 2×, Dorzolamide 2×, Ketorolac 2×, Lotemax® 1×; OS: Dorzolamide 2×, Ketorolac 2×, Lotemax® 1× |
| **September 2021** | 0.1 / 0.1 (1.0 / 1.0 logMAR) | 18 / 16 mmHg | Stable ophthalmologic status | Interferon-beta 1b (Betaferon®) 3×/week; Decortin H alternating 7.5/5 mg/day; Pantoprazole 20 mg/day | Bilateral: Dorzolamide 2×, Ketorolac 2×, Lotemax® 1×; OD: Brimonidine 2× |
| **June 2022** | 0.1 / 0.1 (1.0 / 1.0 logMAR) | 9 / 7 mmHg | New anterior chamber inflammation (OD>OS) | Decortin H 20 mg/day → biweekly taper; Interferon-beta 1b 3×/week; Pantoprazole 20 mg/day | Bilateral: Dorzolamide 2×, Ketorolac 2×, Lotemax® 5×; OD: Brimonidine 2× |
| **July 2022** | 0.05 / 0.1 (1.3 / 1.0 logMAR) | 17 / 13 mmHg | Persistent intraocular inflammation; SD-OCT: OD fovea dry, OS central macular edema regressing | Decortin H 12.5 mg/day → taper; Interferon-beta 1b 3×/week 🡪 discontinued and replaced with azathioprine  Pantoprazole 20 mg/day | Bilateral: Dorzolamide 2×, Ketorolac 2×, Lotemax® 5×; OD: Brimonidine 2× |
| **October 2022** | 0.1 / 0.1 (1.0 / 1.0 logMAR) | 16 / 13 mmHg | Stable panuveitis | Decortin H 7.5 mg/day; Azathioprine 50 mg/day → weekly increase; Pantoprazole 20 mg/day | OD: Brimonidine 2×, Dorzolamide 2×, Ketorolac 2×, Lotemax® 5×; OS: Dorzolamide 2×, Ketorolac 2×, Lotemax® 5×; Betaferon® discontinued |
| **February 2023** | 0.1 / 0.1 (1.0 / 1.0 logMAR) | 13 / 11 mmHg | Increased intraretinal fluid bilaterally | Decortin H 7.5 mg/day; Azathioprine 100 mg 2×/day | OD: Brimonidine 2×, Dorzolamide 2×, Nevanac 3% 2→1×, Lotemax® taper; OS: Dorzolamide 2×, Nevanac 3% 2→1×, Lotemax® taper |
| **August 2023** | 0.1 / 0.1 (1.0 / 1.0 logMAR) | 13 / 11 mmHg | Stable findings | Same as Feb 2023 | Same as Feb 2023 |
| **December 2023** | 0.1 / 0.25 (1.0 / 0.6 logMAR) | 15 / 10 mmHg | Stable panuveitis | Decortin H 7.5 mg/day; Azathioprine 100 mg 2×/day | OD: Dorzolamide 2×, Nevanac 1×, Lotemax® 5×, Brimonidine 2×; OS: Dorzolamide 2×, Nevanac 1×, Lotemax® 5× |
| **May 2024** | 0.125 / 0.1 (0.9 / 1.0 logMAR) | 13 / 11 mmHg | SD-OCT: OD fovea dry, single cyst, subretinal fluid present; OS fovea dry, minimal central macular edema, subretinal fluid slightly increased | Decortin H 7.5 mg/day; Azathioprine 100 mg 2×/day | OD: Dorzolamide 2×, Nevanac 1×, Lotemax® 5×, Brimonidine 2×; OS: Dorzolamide 2×, Nevanac 1×, Lotemax® 5× |
| **July 2024** | not specified | not specified | not specified | Azathioprine discontinued, Ofatumumab (Kesimpta®): 17 July 2024 initiated | not specified |
| **November 2024** | 0.1 / 0.1 (1.0 / 1.0 logMAR) | 10 / 11 mmHg | Mild bilateral inflammatory activity; OCT stable | Decortin H 7.5 mg/day; Ofatumumab 1×/month | OD: Lotemax tapered 5→1×, Dorzolamide 2×, Nevanac 1×, Brimonidine 2×; OS: Lotemax tapered 5→1×, Dorzolamide 2×, Nevanac 1× |
| **March 2025** | 0.05 / ~0.2 (1.3 / 0.7 logMAR) | not specified | Stable visual impairment | Ofatumumab 1×/month | not specified |
| **June 2025** | 0.1 / 0.2 (1.0 / 0.7 logMAR) | 13 / 11 mmHg | Stable ophthalmologic status; FA: diffuse capillary leakage, no active vasculitis | Ofatumumab 1×/month; Decortin H 7.5 mg/day | not specified |

Abbreviations: MRI: Magnetic Resonance Imaging, OD: oculus dexter, OS: oculus sinister, SD-OCT: Spectral-domain optical coherence tomography, FA: Fluorescein angiography
